# Pre-clinical evaluation of novel anti-tuberculosis molecules

**DOI:** 10.1186/1753-6561-8-S4-O17

**Published:** 2014-10-01

**Authors:** Maria Martha Campos

**Affiliations:** 1 PUCRS - Pontificia Universidade do Rio Grande do Sul, Porto Alegre, Brazil

## 

Tuberculosis (TB) currently represents a major global health concern, especially when considering the emergence of multidrug-resistant strains of *Mycobacterium tuberculosis*. On this regard, the identification of new effective agents to treat this infectious disease is urgently needed. Herein, we describe the pre-clinical assays on the anti-tubercular effects of a pentacyano(isoniazid)ferrate(II) compound, namely IQG-607. Previous experimental evidence [1] clearly demonstrated the *in vivo *effectiveness of this compound in a mouse model of TB infection. Accordingly, IQG-607 (22 to and 560 µmol/kg) was able to markedly reduce the number of colony-forming units (CFU) in both spleen and lungs of *M. tuberculosis* H37Rv-infected animals, following long-term schemes of oral administration, during 28 or 56 days. In this series of experiments, the effects of IQG-607 were comparable to those observed for the reference compound isoniazid (182 µmol/kg). More recently, we demonstrated that *in vitro* incubation of IQG-607 led to a marked reduction of CFU counts in *M. tuberculosis* H37Rv-infected macrophages, being this effect similar to that displayed by either isoniazid or rifampicin [2]. Concerning the mechanism of action, IQG-607 was found able to block the biosynthesis of mycolic acids, as indicated by radiolabelling studies with acetate incorporation [2], and it displayed bactericidal effects when tested *in vivo*[1]. The acute oral administration of IQG-607 (560 to 1120 µmol/kg) was not related to any clinical sign of toxicity, whereas the treatment with isoniazid (182 µmol/kg) led to death rates of 80% in mice [3]. The same doses of IQG-607 were also devoid of toxicity, when administered acutely by oral route to rats (unpublished data). To extend evidence on the safety profile of IQG-607, this molecule was also examined in a protocol of repeated dose 90-day oral toxicity in rats. As a main conclusion, there was no evidence of severe toxic signals, although it was not possible to accurately define the NOAEL (non-toxic adverse effect level) for IQG-607. Nevertheless, the dose of 56 µmol/kg might be assumed, once the animals treated with this dose did not show any signal of toxicity (unpublished data). Taken together, our results provide convincing evidence on the efficacy of IQG-607. It can be assumed that IQG-607 is well absorbed when dosed by oral route, being able to reach the bacilli and killing them within the phagosome of the macrophages. Furthermore, we demonstrated a favorable toxicological outcome for this compound, even when dosed acutely, or in long-term schedules of administration, for both mice and rats. Further studies are required to determine the safety of IQG-607 in non-rodent species, as well as the efficacy of this molecule against resistant strains of *M. tuberculosis*. Although additional experiments with this molecule are still required, it is tempting to infer that IQG-607 might well represent a lead compound for development of innovative anti-TB drugs.
